# Antileishmanial Efficacy of the Calpain Inhibitor MDL28170 in Combination with Amphotericin B

**DOI:** 10.3390/tropicalmed7020029

**Published:** 2022-02-16

**Authors:** Marta H. Branquinha, Pedro S. S. Araújo, Simone S. C. Oliveira, Leandro S. Sangenito, Diego S. Gonçalves, Sérgio H. Seabra, Claudia M. d’Avila-Levy, André L. S. Santos

**Affiliations:** 1 Laboratório de Estudos Avançados de Microrganismos Emergentes e Resistentes, Departamento de Microbiologia Geral, Instituto de Microbiologia Paulo de Góes, Universidade Federal do Rio de Janeiro (UFRJ), Rio de Janeiro 21941-902, Brazil; pedro_soares1984@hotmail.com (P.S.S.A.); simonesantiagorj@yahoo.com.br (S.S.C.O.); ibastefano@hotmail.com (L.S.S.); fusariumsp@gmail.com (D.S.G.); 2 Programa de Pós-Graduação em Bioquímica, Instituto de Química, Universidade Federal do Rio de Janeiro (UFRJ), Rio de Janeiro 21941-902, Brazil; 3 Laboratório de Biologia Celular e Tecidual, Centro de Biociências e Biotecnologia, Universidade Estadual do Norte Fluminense Darcy Ribeiro, Campos dos Goytacazes 28013-602, Brazil; seabrash@gmail.com; 4 Laboratório de Estudos Integrados em Protozoologia, Instituto Oswaldo Cruz, Fundação Oswaldo Cruz, Rio de Janeiro 21040-900, Brazil; davila.levy@gmail.com

**Keywords:** *Leishmaniasis*, chemotherapy, MDL28170, amphotericin B

## Abstract

The necessity of drug combinations to treat leishmaniasis came to the surface mainly because of the toxicity of current treatments and the emergence of resistant strains. The calpain inhibitor MDL28170 has previously shown anti-*Leishmania* activity, therefore its use in association with standard drugs could provide a new alternative for the treatment strategy against leishmaniasis. In this study, we analyzed the potential of the combination of MDL28170 and the antileishmanial drug amphotericin B against *Leishmania amazonensis* and *Leishmania chagasi*. The compounds were tested in the combination of the ½ × IC_50_ value of MDL28170 plus the ¼ × IC_50_ value of amphotericin B, which led to an increment in the anti-promastigote activity when compared to the single drug treatments. This drug association revealed several and severe morphophysiological changes on parasite cells, such as loss of plasma membrane integrity, reduced size of flagellum, and depolarization of mitochondrial membrane potential besides increased reactive oxygen species production. In addition, the combination of both drugs had a deleterious effect on the *Leishmania*–macrophage interaction, reflecting in a significant anti-amastigote action, which achieved a reduction of 50% in the association index. These results indicate that the combination treatment proposed here may represent a new alternative for leishmaniasis chemotherapy.

## 1. Introduction

*Leishmaniasis* is a neglected disease that is caused by over 20 species of protozoan parasites from the *Leishmania* genus. Clinical manifestations can vary from cutaneous localized to severe diffuse tegumentar lesions or to a visceral disease that leads to death in non-treated patients [1,2]. Pentavalent antimonials, such as meglumine antimoniate (Glucantime) and sodium stibogluconate (Pentostan), have been employed as first-line treatment for decades and, in case of resistance to these drugs, amphotericin B (AmB), pentamidine, and miltefosine are used as alternatives [3,4]. Despite these options, treatment of leishmaniasis remains non-satisfactory due to the increase in drug resistance, relapses, length of treatment required, and toxicity of compounds to the host, which culminate in poor patient compliance [5,6]. Furthermore, high costs and lack of access in certain areas also contribute for the ineffectiveness of treatment [2,7]. 

The search for new drugs to treat leishmaniasis is still incipient. Drug development faces many obstacles and new drug candidates against leishmaniasis often did not progress further to clinical trials. The few advances so far are limited to the development of new formulations for clinical available drugs or drug repurposing [3,7]. In this context, combination drug therapy has been increasingly explored, with the purpose of identifying cheaper and shorter treatments, requiring minimal clinical monitoring. Besides, combination therapy may also delay emergence of resistance as well as may rise lifetime of the respective drugs, as it has been used in the treatment of malaria, tuberculosis, and HIV [7]. Under this perspective, many studies aim to identify promising leishmanicidal drug associations [4,7]. 

In the exploration of new targets and compounds against trypanosomatids, our group has started working with the calpain inhibitor MDL28170 and its effects in different aspects of *Leishmania* cell biology and physiology [8,9,10,11]. Calpains are neutral calcium-dependent cysteine peptidases displaying a variety of functions related to physiological and pathological events in humans, and homologues of these proteins have been described in other organisms, including trypanosomatids [12,13,14,15,16]. Our group has previously demonstrated the effect of MDL28170 in promoting cellular alterations and growth arrest in promastigotes of *L. amazonensis* [8]. In this parasite, MDL28170 also induces apoptotic marker expression in promastigote forms [9] and it was able to reduce the interaction process of promastigotes with macrophages [10]. The calpain inhibitor also diminished the infection rate, presenting IC_50_ values for amastigotes in the same range as promastigotes [10]. In addition, the susceptibility of six *Leishmania* species (*L. amazonensis*, *L. braziliensis*, *L. chagasi*, *L. donovani*, *L. major* and *L. mexicana*) to MDL28170 was compared: promastigote proliferation and the number of intracellular amastigotes in RAW macrophages were reduced for all the parasites tested, displaying high selectivity index values [11].

Since MDL28170 has been shown to be effective against distinct *Leishmania* spp., its use in combination with standard drugs could provide new alternatives for the treatment of leishmaniasis. The objective of the present study was to determine the potential of the combination therapy using this calpain inhibitor associated with AmB against both evolutive forms of *L. amazonensis* and *L. chagasi,* relevant causative agents of cutaneous and visceral forms of leishmaniasis, respectively, in Brazil. Altogether, the interaction of MDL28170 and AmB has revealed important biological consequences against *L. amazonensis* and *L. chagasi.*

## 2. Materials and Methods

### 2.1. Parasites and Culture

Promastigotes of *L. amazonensis* (MHOM/BR/PH8) and *L. chagasi* (MHOM/BR/1974/PP75) were obtained from *Leishmania* Type Culture Collection-LTTC-WDCM 731 (Instituto Oswaldo Cruz, Fundação Oswaldo Cruz, Rio de Janeiro, Brazil) and routinely cultured in Schneider’s medium, pH 7.2, supplemented with 10% heat-inactivated fetal bovine serum (FBS) at 28 °C.

### 2.2. Antileishmanial Compounds

Calbiochem (San Diego, CA, USA) supplied the cell-permeable calpain inhibitor employed in this study: MDL28170 (Z-Val-Phe-CHO; Z = *N*-benzyloxycarbonyl) is a reversible peptidomimetic inhibitor, also known as calpain inhibitor III. The stock solution was prepared in dimethylsulfoxide (DMSO; Sigma-Aldrich, Saint Louis, MO, USA) at 5 mM. AmB was purchased from Sigma-Aldrich, and a 20 mg/mL stock solution was prepared in water.

### 2.3. Effect of AmB on the Growth Rate of Promastigotes

The effect of AmB on promastigotes of *L. amazonensis* and *L. chagasi* was assessed by a method similar to that described previously [8]. Promastigotes were counted using a Neubauer chamber and resuspended in fresh medium at a final concentration of 5 × 10^5^ viable promastigotes/mL. AmB was added to the culture at final concentrations ranging from 0.005 µg/mL to 0.04 µg/mL. After 24, 48, 72, and 96 h of incubation at 28 °C, the number of parasites was estimated daily by counting the flagellates in a Neubauer chamber, in which viability was assessed by mobility and lack of staining after challenging with Trypan blue. The 50% inhibitory concentration (IC_50_), i.e., the minimum drug concentration that caused a 50% of reduction in survival/viability was determined after 48 h by non-linear regression, plotting the log number of promastigotes versus drug concentration using Microsoft Excel software.

### 2.4. Combination of the Calpain Inhibitor MDL28170 and AmB

After establishing the IC_50_/48 h value of AmB for both species and with the IC_50_/48 h values of MDL28170 previously determined for *L. amazonensis* and *L. chagasi* by our group [11] as 6.0 and 8.1 µM, respectively, we performed combination assays of these drugs in 24-well plates. For this, promastigotes (5 × 10^5^ viable cells/mL) were incubated in the presence of the ⅛, ¼ or ½ × IC_50_/48 h values of AmB and the simultaneous addition of the ⅛, ¼ or ½ × IC_50_/48 h values of the calpain inhibitor. The number of viable motile parasites was estimated daily by counting the flagellates in a Neubauer chamber, as described previously.

From this point on, the experiments described in topics 2.5–2.9 were performed with parasites (5×10^5^ viable cells/mL) incubated in the following systems for 48 h: (i) control cells (grown in the absence of any compound); (ii) cells incubated with the drugs in the combination of AmB at the ¼ × IC_50_/48 h value and MDL28170 at the ½ × IC_50_/48 h value; (iii) cells incubated with AmB at the ¼ × IC_50_/48 h value; and (iv) cells incubated with MDL28170 at the ½ × IC_50_/48 h value.

### 2.5. Scanning Electron Microscopy (SEM)

Promastigotes of *L. amazonensis* and *L. chagasi* incubated in the different systems described previously were fixed in cacodylate buffer 0.1 M (pH 7.2) containing formaldehyde 4%, glutaraldehyde 2.5%, and CaCl_2_ 5 mM for 1 h at room temperature. After this step, cells were washed with cacodylate buffer and added to glass coverslips in 24-well plates containing 75 µL of poly-L-lysine previously incubated for 1 h at 37 °C. Then, cells were covered with osmium tetroxide 1% and cacolylate buffer 0.1 M on a 1:1 ratio for 30 min in the dark. Subsequently, promastigotes were washed 3 times in PBS (phosphate buffered saline, pH 7.2) and dehydrated in rising acetone concentrations (30, 50 and 70%) for 15 min each. Cells were dried on critical point and metallized with gold (25 nm) at 40 mA for 160 s and then observed in a JEOL JSM 649-OLV scanning electronic microscope [17].

### 2.6. Cell Membrane Integrity

Promastigotes of *L. amazonensis* and *L. chagasi* incubated in the different systems described previously were washed in PBS (pH 7.2) and incubated with 10 μM propidium iodide (PI), a DNA-binding vital dye, for 30 min at room temperature, protected from light. The parasites were washed in PBS and their fluorescence was quantified on a flow cytometer (FACSCalibur, BD Biosciences) equipped with a 15-mW argon laser emitting at 535 nm. The mapped population (10,000 events) was analyzed for log red fluorescence using a single-parameter histogram, and the results were expressed as the percentage of fluorescent cells (%FC). Autoclaved cells were used as a positive control of passive PI incorporation.

### 2.7. Determination of Mitochondrial Transmembrane Electric Potential

The mitochondrial transmembrane electric potential (ΔΨm) was investigated using the JC-1 fluorochrome (Sigma-Aldrich), which is a lipophilic cationic mitochondrial vital dye that becomes concentrated in the mitochondrion in response to ΔΨm. Thus, the fluorescence of JC-1 is considered an indicator of an energized mitochondrial state, and it has been used to measure the ΔΨm in *Leishmania* [18]. Promastigotes of *L. amazonensis* and *L. chagasi* incubated in the different systems described previously were harvested after 48 h, washed with PBS and added to a reaction medium containing 125 mM sucrose, 65 mM KCl, 10 mM HEPES/K^+^, pH 7.2, 2 mM Pi, 1 mM MgCl_2_ and 500 µM EGTA. To evaluate the ΔΨm for each experimental condition, 2 × 10^7^ parasites were incubated with 10 µg/mL JC-1 for 40 min, with readings made every minute using a microplate reader (SpectraMax spectrofluorometer, Molecular Devices). The relative ΔΨm value was obtained by calculating the ratio between the reading at 590 nm and the reading at 530 nm (590:530 ratio) using an excitation wavelength of 480 nm. Cells were also incubated in the presence of carbonyl cyanide 4-(trifluoromethoxy)phenylhydrazone (FCCP) at 1 µM, a mitochondrial protonophore, as a positive control of the depolarization of mitochondrial membrane. FCCP at the concentration of 2 µM was also added at the end of all experiments to abolish ΔΨm. This allowed comparison of the magnitude of ΔΨm under the different experimental conditions.

### 2.8. ROS Production

Promastigotes of *L. amazonensis* and *L. chagasi* incubated in the different systems described previously were washed in PBS, suspended in 500 µL of PBS, and incubated with the cell-permeable probe dichlorofluorescein (H_2_DCFDA) at 40 µM for 30 min in the dark. Then, cells were harvested at 500× *g*/5 min, resuspended in PBS, and analyzed by flow cytometry (FACSCalibur, BD Biosciences). Cells treated with hydrogen peroxide at 17 mM were used as a positive control [19]. The mapped population (10,000 events) was analyzed for log green fluorescence using a single-parameter histogram, and the results were expressed as the %FC.

### 2.9. Leishmania–Macrophage Interaction

RAW 264.7 macrophages were placed in a 24-well plate at a ratio of 5 × 10^4^ macrophages per well in 13-mm glass coverslips and incubated at 37 °C in Dulbecco’s modified Eagle’s medium (DMEM) supplemented with 10% FBS in an atmosphere containing 5% CO_2_. *L. amazonensis* and *L. chagasi* (5 × 10^5^ promastigotes) were washed with PBS and pre-treated or not with each system described previously for 4 h, the time interval in which more than 95% of the parasites were viable, as judged by their morphology and motility (data not shown). Macrophages were then infected with promastigote forms at a ratio of 10:1 (parasites:macrophage) for 24 h at 37 °C in a 5% CO_2_ atmosphere, after which macrophage monolayers were washed with PBS to remove unbound parasites. The coverslips were then fixed in methanol, stained with Giemsa and dehydrated in acetone solutions progressively replaced by xylol. The percentage of infected macrophages was determined by randomly counting at least 200 cells in each of duplicated coverslips. The association index was obtained by multiplying the percentage of infected macrophages by the number of amastigotes per infected macrophage [20].

### 2.10. Intracellular Amastigotes Viability

For this purpose, RAW macrophages were plated as described previously and then infected with promastigote forms of *L. amazonensis* and *L. chagasi* (10 parasites per each mammalian cell) for 24 h at 37 °C, 5% CO_2_ to allow parasite internalization. Afterwards, the cultures were washed with sterile PBS to remove non-internalized parasites and then fresh DMEM medium with 10% FBS was added. Infected macrophages were treated or not with the drugs in the same combination used in promastigotes, in which AmB was used at the ¼ × IC_50_ value and MDL28170 at the ½ × IC_50_ value; however, these were determined for intracellular amastigotes prior to the interaction assay. The IC_50_ values of MDL28170 for intracellular amastigotes were previously determined as 4.9 and 7.0 µM for *L. amazonensis* and *L. chagasi,* respectively [11], and the IC_50_ values of AmB for intracellular amastigotes after 48 h were determined as 0.0077 µg/mL (*L. amazonensis*) and 0.014 µg/mL (*L. chagasi*) (data not shown). These concentrations in combination were capable of maintaining 95% macrophage viability (data not shown). After 24 h and 48 h of treatment, the interaction systems were washed again with PBS, the slides were fixed in methanol, and stained with Giemsa. The association index was obtained as described previously.

### 2.11. Statistical Analysis

All experiments were performed in triplicate in three independent experimental sets. Data were analyzed statistically using unidirectional ANOVA test using GraphPad Prism 4.0 software (Prism, version 8.0; GraphPad Software; San Diego, CA, USA, 2018). Descriptive analysis, including mean and standard deviation, was used to evaluate numerical data. *p* values of 0.05 or less were considered statistically significant.

## 3. Results

### 3.1. Combination Therapy against Leishmania: MDL28170 and AmB

**Figure A1:**
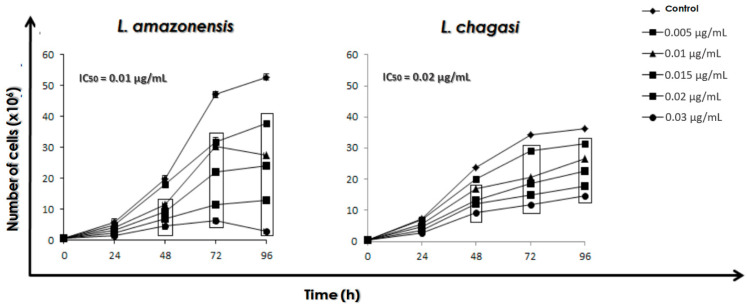
Effects of amphotericin B on the proliferation of promastigotes of *L. amazonensis* and *L. chagasi*. The growth rate of promastigotes was monitored when the parasites were cultured at 28 °C in the absence (control) or in the presence of different concentrations of amphotericin B. The number of viable cells was estimated daily up to 96 h in a Neubauer chamber, and calculated IC_50_ values are indicated. Results represent mean ± standard deviation of three independent experiments performed in triplicate. Boxes highlight significant different values when compared to control (*p* ˂ 0.05).

Initially, promastigotes of both *Leishmania* species were incubated in the absence (control) or in the presence of different concentrations of AmB in order to determine the IC_50_ values for this drug. After 48 h of incubation (exponential growth phase), the IC_50_ values were calculated to be 0.01 µg/mL for *L. amazonensis* and 0.02 µg/mL for *L. chagasi* (Figure A1). As our group has previously established the IC_50_/48 h values of MDL28170 for *L. amazonensis* and *L. chagasi* as 6.0 and 8.1 µM, respectively [11], combination assays were then performed with both compounds, MDL28170 and AmB, for treating promastigotes of *L. amazonensis* and *L. chagasi*. For this, the ⅛, ¼, or ½ × IC_50_/48 h values of each drug were combined and cell proliferation was measured after 48 h.

**Figure 1:**
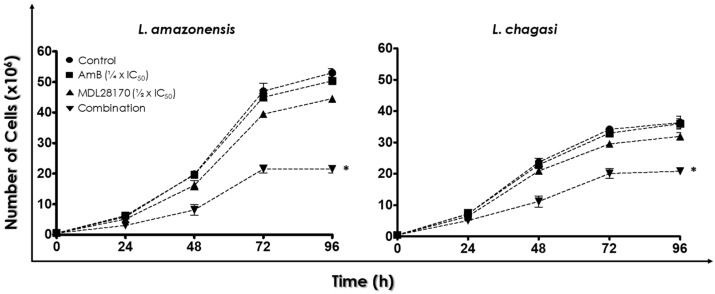
Effects of the combination of ¼ × IC_50_ value of amphotericin B (AmB) plus ½ × IC_50_ value of the calpain inhibitor MDL28170 on promastigotes of *L. amazonensis* and *L. chagasi*. The growth rate of promastigotes was monitored when the parasites were cultured at 28 °C in the absence (control) or in the presence of both drugs in the combination cited above. In addition, growth rate of promastigotes was monitored in the presence of either the ¼ × IC_50_ value of AmB or the ½ × IC_50_ value of MDL28170. The number of viable cells was estimated daily up to 96 h in a Neubauer chamber. Results represent mean ± standard deviation of three independent experiments performed in triplicate. Asterisks mean significant different values when compared to control (*p* < 0.05).

**Figure A2:**
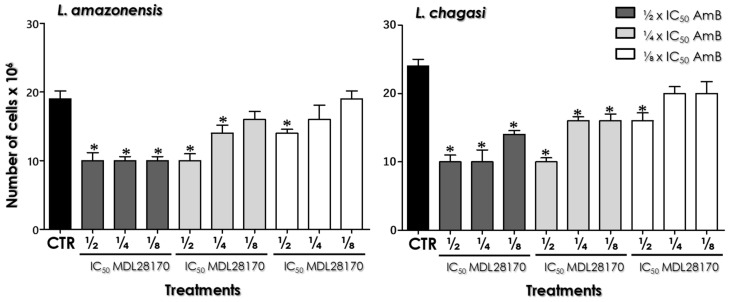
Effects of the combination of Amphotericin B (AmB) and MDL28170 on the growth rate of *L. amazonensis* and *L. chagasi*. Promastigotes were incubated or not (Control, CTR) with combinations of both drugs at the concentrations corresponding to the ⅛, ¼, or ½ × IC_50_/48 h values of each drug and cell proliferation was measured after 48 h. Results represent mean ± standard deviation of three independent experiments performed in triplicate and the asterisks highlight significant differences when compared to control (*p* < 0.05).

The combinations containing the ⅛ × IC_50_ value of AmB were the least effective over the parasites. On the other hand, the combinations including the ½ × IC_50_ value of AmB exhibited the greatest impact over promastigotes, displaying similar values of growth inhibition irrespective of the fractioned value of MDL28170 in association. When the ¼ × IC_50_ value of AmB was employed, the association with the ½ × IC_50_ value of MDL28170 showed similar values of cell proliferation inhibition when compared to the combinations containing the ½ × IC_50_ value of AmB (Figure A2). Considering that the latter combination elicited a similar response with a lowest dose of AmB, the ¼ × IC_50_ value of AmB plus the ½ × IC_50_ value of MDL28170 was elected for performing the following experiments. When the parasites were cultured for 72 h, a time-dependent reduction in the proliferation rate was observed in this association in comparison to control cells as well as in comparison to cells cultured in the presence of either the ¼ × IC_50_ value of AmB or the ½ × IC_50_ value of MDL28170 (Figure 1).

### 3.2. Promastigotes Morphology

**Figure 2:**
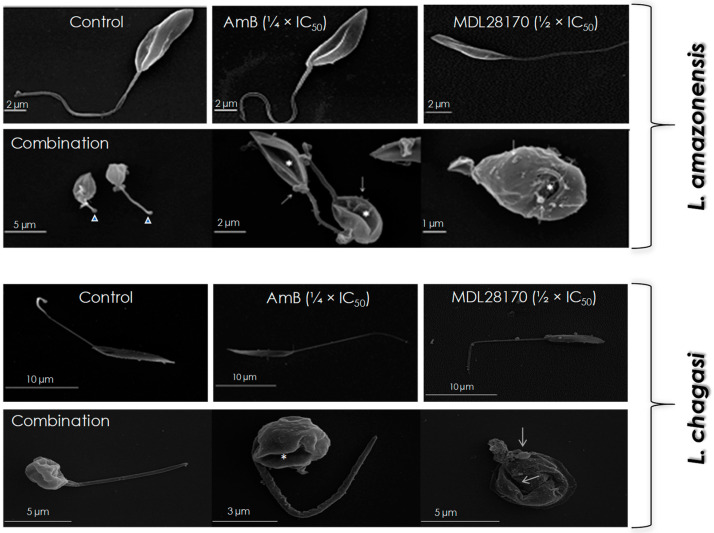
Effects of the combination of ¼ × IC_50_ value of amphotericin B (AmB) plus ½ × IC_50_ value of the calpain inhibitor MDL28170 on the cell morphology of promastigotes of *L. amazonensis* and *L. chagasi*. Parasites were incubated for 48 h in the absence (control) or in the presence of the drugs in combination, and then processed for scanning electron microscopy. Cells were also incubated in the presence of either the ¼ × IC_50_ value of AmB or the ½ × IC_50_ value of MDL28170. Promastigotes treated with the drugs in combination presented altered size, rounding cell shape, reduced size of flagellum (arrowheads), plasma membrane disruption (asterisks), and the formation of blebs (white arrows).

*Leishmania amazonensis* and *L. chagasi* cells were incubated for 48 h in the absence (control) and in the presence of the combination of both drugs (¼ × IC_50_ value of AmB plus ½ × IC_50_ value of MDL28170) in order to evaluate the effects on the promastigote morphology. In addition, parasites were incubated with the same concentrations of each compound separately. When compared to control (untreated) cells, AmB- or MDL28170-treated promastigotes showed no significant morphological alterations in SEM images: cells retained their normal features, like the typical elongated shape and a long flagellum (Figure 2). However, the combination of the ¼ × IC_50_ value of AmB plus the ½ × IC_50_ value of MDL28170 was able to cause significant alterations on the morphology of promastigotes of both parasites, such as altered size, rounding cell shape, and reduced size of flagellum, besides plasma membrane disruption and the formation of blebs (Figure 2).

### 3.3. Plasma Membrane Integrity

**Figure 3:**
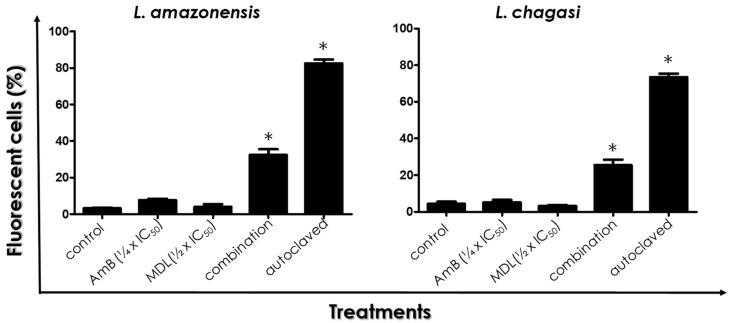
Effects of the combination of ¼ × IC_50_ value of amphotericin B (AmB) plus ½ × IC_50_ value of the calpain inhibitor MDL28170 on the cell membrane integrity of promastigotes of *L. amazonensis* and *L. chagasi*. Parasites were incubated for 48 h in the absence (control) or in the presence of the drugs in combination. Parasites were also incubated in the presence of either the ¼ × IC_50_ value of AmB or the ½ × IC_50_ value of MDL28170. Then, cells were incubated with propidium iodide and evaluated by flow cytometry. Autoclaved cells were used as positive control. Results are expressed as the percentage of fluorescent cells in each system. The plotted data are mean ± standard error of three independent experiments performed in triplicate. The asterisks highlight significant different values when compared to control (*p* < 0.05).

Promastigotes of both species treated in the absence or in the presence of the drugs in combination or separately were incubated with PI and analyzed by flow cytometry. For *L. amazonensis* and *L. chagasi*, no significant difference was found in the %FC among control (untreated) parasites and promastigotes treated with either the ¼ × IC_50_ value of AmB or the ½ × IC_50_ value of MDL28170 (Figure 3). However, drugs in combination led to an 8-fold and 6.6-fold increase in the percentage of PI-labeled promastigotes of *L. amazonensis* and *L. chagasi*, respectively, showing a significant loss in plasma membrane integrity (Figure 3). Autoclaved cells were used as positive controls and, as expected, displayed an elevated number of fluorescent cells, which indicated a significant passive incorporation of PI (Figure 3).

### 3.4. ΔΨ m Measurements

In order to evaluate alterations in the parasite mitochondrion, which is a unique and vital organelle, promastigotes of both *Leishmania* species were incubated in the absence (control) or in the presence of the drugs in combination or separately at the same concentrations for 48 h. After this period, parasites were processed as described previously, using JC-1.

**Figure 4:**
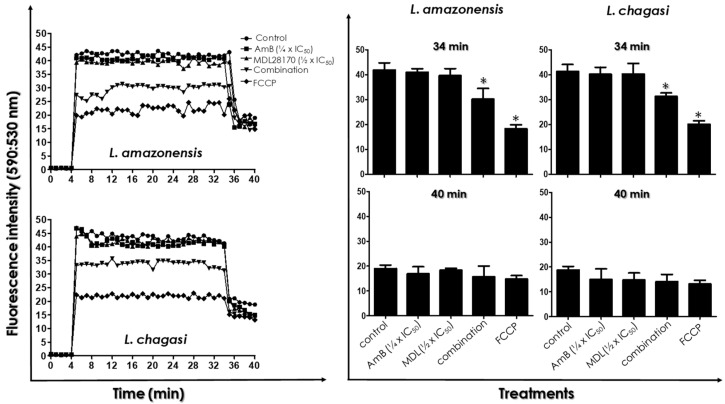
Effects of the combination of ¼ × IC_50_ value of amphotericin B (AmB) plus ½ × IC_50_ value of the calpain inhibitor MDL28170 on the ΔΨm in promastigotes of *L. amazonensis* and *L. chagasi*. Parasites were incubated for 48 h in the absence (control) or in the presence of the drugs in combination. Parasites were also incubated in the presence of either the ¼ × IC_50_ value of AmB or the ½ × IC_50_ value of MDL28170. After this period, ΔΨm was evaluated by the addition of JC-1 fluorochrome for 30 min with readings every minute (left panels). After 34 min, the uncoupler FCCP was added to all systems at 2 µM to collapse mitochondrial potential. As a positive control of the depolarization of the mitochondrial membrane, the reaction was also evaluated in the presence of FCCP (1 μM) from the beginning of the experiment. In the right, graphs show the values before (34 min) and after the addition of FCCP (40 min). Results are expressed as fluorescence intensity by calculating the ratio between the reading at 590 nm and the reading at 530 nm (590:530 nm). The plotted data are mean ± standard error of three independent experiments performed in triplicate. The asterisks highlight significant different values when compared to control (*p* < 0.05).

After JC-1 treatment, no significant difference was found in the ΔΨm for the two studied parasites among control (untreated) cells and promastigotes treated with either the ¼ × IC_50_ value of AmB or the ½ × IC_50_ value of MDL28170 (Figure 4). On the other hand, treatment of parasites with the drugs in combination led to a significant reduction of ΔΨm when compared with control cells and to the single drug treatments (Figure 4). In addition, pre-incubation with FCCP resulted in decreased mitochondrial staining with JC-1. At 34 min of reaction, FCCP reduced the ΔΨm in 53–57%, while the drugs in combination induced the mitochondrial depolarization by 27% in *L. amazonensis* and by 24% in *L. chagasi.* After 34 min of JC-1 uptake, the addition of 2 µM FCCP fully collapsed the ΔΨm, including control parasites (Figure 4).

### 3.5. ROS Production

**Figure 5:**
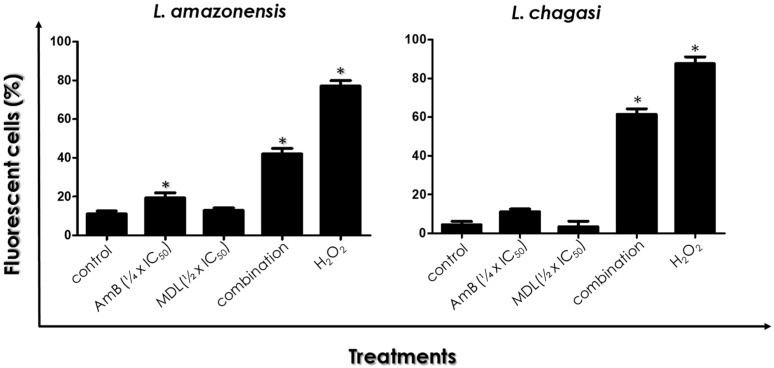
Effects of the combination of ¼ × IC_50_ value of amphotericin B (AmB) plus ½ × IC_50_ value of the calpain inhibitor MDL28170 on ROS production by promastigotes of *L. amazonensis* and *L. chagasi*. Parasites were incubated for 48 h in the absence (control) or in the presence of the drugs in combination. Parasites were also incubated in the presence of either the ¼ × IC_50_ value of AmB or the ½ × IC_50_ value of MDL28170. After treatment, cells were incubated with H_2_DCFDA for evaluation of ROS production. Results are expressed as the percentage of fluorescent cells in each system. The plotted data are mean ± standard error of three independent experiments performed in triplicate. The asterisks highlight significant different values when compared to control (*p* < 0.05).

Promastigotes of both Leishmania species incubated in the absence (control) or in the presence of drugs in combination or separately at the same concentrations were processed as previously described for ROS quantification. *L. amazonensis* and *L. chagasi* promastigotes treated with the ½ × IC_50_ value of MDL28170 showed no significant alteration in ROS production in comparison to control cells (Figure 5). When cells were treated with either the ¼ × IC_50_ value of AmB or with the drugs in combination, an increase in ROS production was detected: a 2-fold and 3-fold increase in the %FC of *L. amazonensis* and *L. chagasi*, respectively, after treatment with the ¼ × IC_50_ value of AmB; and a 4-fold and 17-fold increase in the %FC of *L. amazonensis* and *L. chagasi*, respectively, after treatment with the drugs in combination (Figure 5). Cells treated with H_2_O_2_ were used as positive controls and displayed high %FC (Figure 5).

### 3.6. Leishmania–Macrophage Interaction: Pre-Treatment

**Figure 6:**
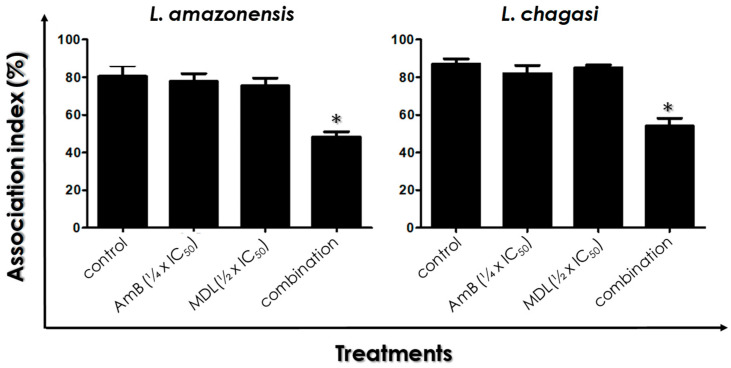
Effects of the combination of ¼ × IC_50_ value of amphotericin B (AmB) plus ½ × IC_50_ value of the calpain inhibitor MDL28170 on *L. amazonensis* and *L. chagasi* promastigotes interaction with macrophages. Promastigotes were pre-treated or not (control) with the drugs in combination for 4 h prior to macrophage–parasite interaction. Macrophages were then infected with promastigote forms for 24 h at 37 °C, and macrophage monolayers were washed with PBS to remove unbound parasites. The association index was determined as described in Materials and Methods. Parasites were also incubated in the presence of either the ¼ × IC_50_ value of AmB or the ½ × IC_50_ value of MDL28170 prior to Leishmania–Macrophage interaction. The results represent the mean and standard deviation of three independent experiments and the asterisks highlight significant differences when compared to control (*p* < 0.05).

Before macrophage infection, *L. amazonensis* and *L. chagasi* promastigotes were treated for 4 h with the drugs in combination or separately. After 24 h of Leishmania–Macrophage interaction, the results showed that the association index was not significantly altered when each compound was used separately, in comparison to control infected macrophage cells (Figure 6). However, treatment with drugs in the combination of the ¼ × IC_50_ value of AmB plus the ½ × IC_50_ value of MDL28170 led to a significant reduction in the association index, by approximately 45% for *L. amazonensis* and by 35% for *L. chagasi* (Figure 6).

### 3.7. Leishmania–Macrophage Interaction: Post-Treatment

**Figure 7:**
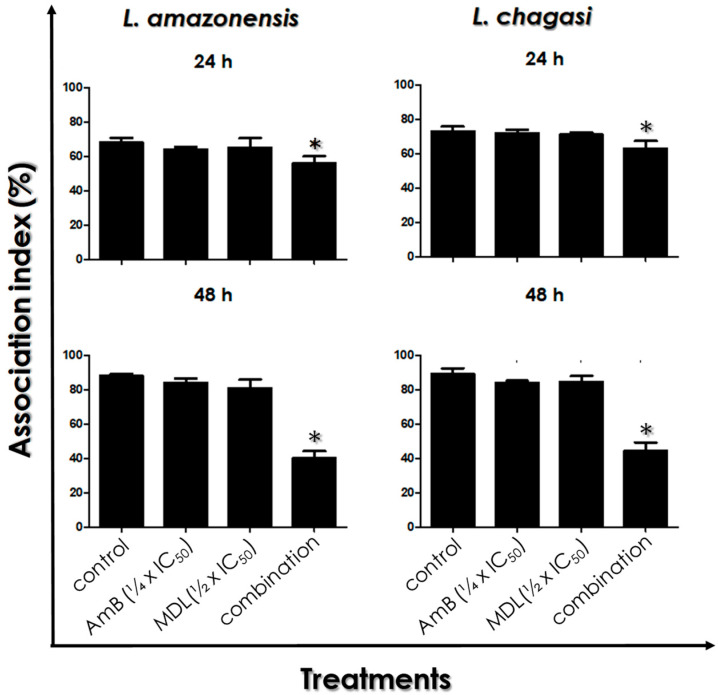
Susceptibility of *L. amazonensis* and *L. chagasi* intracellular amastigotes to the combination of ¼ × IC_50_ value of amphotericin B (AmB) plus ½ × IC_50_ value of the calpain inhibitor MDL28170. Initially, host cells were infected with promastigote forms for 24 h at 37 °C, followed by exhaustive washing in PBS, and then the interaction process was followed at daily intervals up to 48 h in the absence (control) or in the presence of the drugs in combination. Infected host cells were also incubated in the presence of either the ¼ × IC_50_ value of AmB or the ½ × IC_50_ value of MDL28170. The association index was determined as previously described. The results represent the mean and standard deviation of three independent experiments and the asterisks highlight significant differences when compared to control (*p* < 0.05).

In this set of experiments, infected macrophages were treated or not with the ¼ × IC_50_ value of AmB plus the ½ × IC_50_ value of MDL28170 previously determined [11] in order to evaluate the efficacy on the intracellular amastigotes. After 24 and 48 h of treatment, the results showed a time-dependent action of the combination of AmB and MDL28170 in diminishing the viability of intracellular amastigotes of *L. amazonensis* and *L. chagasi*. After 24 h of infection, the drug combination was able to significantly reduce the association index by approximately 20% for both parasites, which was not observed when each drug was used separately (Figure 7). In addition, the 48-h treatment of the infected macrophages promoted a reduction of around 50% in the association index for both Leishmania species. In this time interval, the use of either AmB at the ¼ × IC_50_ value or MDL28170 at the ½ × IC_50_ value did not promote significant alterations in the survival of intracellular amastigotes (Figure 7).

## 4. Discussion

In the past 75 years, the therapeutic arsenal for the treatment of leishmaniasis has been extremely limited due to the increasing numbers of resistance and toxicity, pointing toward the need of novel drug options [6,7].

Consequently, drug combinations for the treatment of leishmaniasis could have a role in delaying the development of resistance, increasing efficacy and shortening the duration of treatment that could improve compliance and reduce cost [6,7]. This approach represents a promising and challenging chemotherapeutic strategy that has been implemented in different endemic areas for leishmaniasis [7,21]. With the purpose to improve the anti-*Leishmania* effect of MDL28170 [8,9,10,11], its in vitro activity, when used in combination with AmB, was explored in the present work with the goal of finding a new possible alternative for the treatment of leishmaniasis.

Combination therapy using drugs with different chemical structures and mechanisms of action have been appointed as a promising strategy to improve actual treatment against leishmaniasis [3,4,5,6,7,22,23]. In this regard, the second-line drug AmB is already consolidated in leishmaniasis treatment, and its major mechanism of action is through binding to sterols that changes the permeability of the plasma membrane and by generating free radicals [24]. However, AmB presents high nephrotoxicity and thus its use is only recommended in cases of low therapeutic response or resistance to antimonials [3]. In the last years, efforts have been performed to improve AmB chemotherapy. Several studies added further information on its mechanisms of action, new methods of preparation, and potential incorporation into different formulations [25,26]. In this context, a promising strategy is the association of AmB with compounds that showed relevant antileishmanial activity, such as calpain inhibitors; such association could reduce drug doses and toxicity of AmB, thereby decreasing both incidence of adverse effects and costs [2,7]. In addition, a combination of two drugs with rather different mechanisms of action over the parasites may have the benefit of hindering the emergence of resistant strains [3,4,5,6,7].

The mechanisms of action of the calpain inhibitor MDL28170 are not properly understood yet. Besides the pleiotropic effects of the calpain inhibitor MDL28170 in *Leishmania* spp. including growth arrest, host cell interaction, and apoptotic markers expression [8,9,10,11], our group has also published that MDL28170 has effect in crucial steps of *T. cruzi* life cycle, such as the interaction with the gut of the invertebrate host and with macrophages, as well as in metacyclogenesis [27,28]. Suggested targets of this calpain inhibitor observed recently in the tomato parasite *Phytomonas serpens* include: mitochondrial swelling, disruption of *trans*-Golgi network, inhibition of processing of cruzipain-like molecules, and decreased interaction with explanted salivary glands of the insect *Oncopeltus fasciatus* [29].

With these premises in mind, combination assays were performed in the present work with both compounds, MDL28170 and AmB, for *L. amazonensis* and *L. chagasi* promastigotes viability, and the association of the ¼ × IC_50_ value of AmB and the ½ × IC_50_ value of MDL28170 was settled as the most effective among the ones tested with the lowest dosage of AmB. The effects of MDL28170 and AmB in combination against *L. amazonensis* and *L. chagasi* promastigotes were accompanied by altered morphology, damages in plasma membrane (the major target of AmB), flagellum shortening, depolarization of mitochondrial electrochemical potential and ROS production (cellular targets of both MDL28170 and AmB), concluding that these drugs affect significantly *Leishmania* metabolism and morphology. The drugs were tested alone (at a concentration equivalent to ¼ × IC_50_ value of AmB or the ½ × IC_50_ value of MDL28170) or in the combination of these fractions. The combination exhibited an impact over promastigotes’ morphology and metabolism, and the degree of alterations apparently indicates an additive interaction of both compounds. This set of results established the feasibility of proceeding to macrophage infection experiments, aiming to analyze the effects of combination treatments on the interaction with host cells and in intracellular amastigotes. The association of AmB and MDL28170 reduced the interaction process of promastigotes with macrophages and, relevantly, reduced intracellular amastigote numbers, measured by the association index, when compared to the use of these compounds individually.

The results presented herein raised the question as to whether the combination of MDL28170 and AmB should be effective against different *Leishmania* spp. In a previous work from our group [11], we showed that six *Leishmania* species, including *L. amazonensis* and *L. chagasi*, presented susceptibility to MDL28170 in a low range of IC_50_ values, varying in promastigote forms from 4 µM in *L. braziliensis* (the most sensitive) to 9 µM in *L. mexicana* (the most resistant), while in amastigote forms, IC_50_ values differed between 2.8 µM and 8.5 µM in the same species, respectively. The intrinsic variation in MDL28170 sensitivity of each *Leishmania* species, although in a similar range, is not surprising, since different biochemical and molecular characteristics are displayed by each member of this genus [30]. Another also relevant aspect is the fact that no correlation in MDL28170 responsiveness with regard to the type of leishmaniasis (cutaneous or visceral) these species cause was found. In conclusion, it is expected that combination of MDL28170 with AmB may present similar repercussions in distinct *Leishmania* spp., as observed in the present work to *L. amazonensis* and *L. chagasi*, but this must be confirmed subsequently.

Several calpain inhibitors were reported in the last years, and their potential therapeutic use is explored for the treatment of several human pathophysiological events in which calpains have been implicated [31]. Differences in the degree of inhibition of calpain activity might be explained by differences in the chemical structure, mechanisms of action, or specificity of calpain inhibitors for a particular calpain structure [31]. This is an important issue, especially for invertebrates and lower eukaryotes such as trypanosomatids displaying non-typical calpains, many of them probably with no proteolytic activity [12,13,14,15,16]. It would be worthwhile to further study the action of these calpain inhibitors against trypanosomatids in the hope that they can give us some insights into their mechanisms of action.

In the present paper, we devised a new approach for leishmaniasis chemotherapy based on the combination treatment of AmB with MDL28170. From these results, it is clear that these drugs in combination revealed the importance of exploring novel classes of bioactive compounds such as calpain inhibitors and demonstrated that they can act with currently used anti-leishmanial drugs, and may improve the therapeutic outcome. However, drug combinations must be used with care given to the possibility that, if not applied in a controlled and regulated way, resistance could be induced in *Leishmania*, thus resulting in a rapid loss of efficacy of not one, but two therapeutic options [32]. Considering the in vitro anti-leishmanial activity of MDL28170 and the effects of AmB and MDL28170 in combination against promastigotes and amastigotes, further in vivo experiments could be useful when considering the interaction between calpain inhibitors and AmB for future recommendations to combat different *Leishmania* infections.
